# Spatio-temporal distribution patterns of *Plutella xylostella* (Lepidoptera: Plutellidae) in a fine-scale agricultural landscape based on geostatistical analysis

**DOI:** 10.1038/s41598-021-92562-9

**Published:** 2021-06-30

**Authors:** Jian-Yu Li, Yan-Ting Chen, Meng-Zhu Shi, Jian-Wei Li, Rui-Bin Xu, Gabor Pozsgai, Min-Sheng You

**Affiliations:** 1 State Key Laboratory of Ecological Pest Control for Fujian and Taiwan Crops, Institute of Applied Ecology, Fujian Agriculture and Forestry University, Fuzhou, 350002 China; 2 Fujian Key Laboratory for Monitoring and Integrated Management of Crop Pests, Institute of Plant Protection, Fujian Academy of Agricultural Sciences, Fuzhou, 350013 China; 3 College of Physics and Information Engineering, Fuzhou University, Fuzhou, 350116 China

**Keywords:** Ecology, Behavioural ecology, Population dynamics

## Abstract

A detailed knowledge on the spatial distribution of pests is crucial for predicting population outbreaks or developing control strategies and sustainable management plans. The diamondback moth, *Plutella xylostella*, is one of the most destructive pests of cruciferous crops worldwide. Despite the abundant research on the species’s ecology, little is known about the spatio-temporal pattern of *P*. *xylostella* in an agricultural landscape. Therefore, in this study, the spatial distribution of *P*. *xylostella* was characterized to assess the effect of landscape elements in a fine-scale agricultural landscape by geostatistical analysis. The *P*. *xylostella* adults captured by pheromone-baited traps showed a seasonal pattern of population fluctuation from October 2015 to September 2017, with a marked peak in spring, suggesting that mild temperatures, 15–25 °C, are favorable for *P*. *xylostella*. Geostatistics (GS) correlograms fitted with spherical and Gaussian models showed an aggregated distribution in 21 of the 47 cases interpolation contour maps. This result highlighted that spatial distribution of *P*. *xylostella* was not limited to the *Brassica* vegetable field, but presence was the highest there. Nevertheless, population aggregations also showed a seasonal variation associated with the growing stage of host plants. GS model analysis showed higher abundances in cruciferous fields than in any other patches of the landscape, indicating a strong host plant dependency. We demonstrate that *Brassica* vegetables distribution and growth stage, have dominant impacts on the spatial distribution of *P*. *xylostella* in a fine-scale landscape. This work clarified the spatio-temporal dynamic and distribution patterns of *P*. *xylostella* in an agricultural landscape, and the distribution model developed by geostatistical analysis can provide a scientific basis for precise targeting and localized control of *P*. *xylostella*.

## Introduction

The diamondback moth (DBM), *Plutella xylostella* (L.), is one of the most destructive economic pests of cruciferous vegetables throughout the world. It is estimated to cost US$4–5 billion of the world economy^1^ and US$0.77 billion of the Chinese economy annually^2^. *P*. *xylostella* prefers to feed on plants of the family Cruciferae^3,4^. Many factors contribute to the success of *P*. *xylostella* as a worldwide agricultural pest, including its short generation time, high fecundity, broad range of host plants, seasonal migration behavior, low intraspecific competition, and strong environmental adaptability^3–7^. Seasonal migration, in particular, is one of the important causes that enable the diamondback moth to become a global pest^8–11^. However, most studies focused on the autecology of *P*. *xylostella*^2,5^ and little is known on its spatio-temporal distribution patterns in agricultural landscape.

Many aspects of the population dynamics of *P*. *xylostella* have recently been documented, with most of these studies analyzing the temporal patterns of adult capture data^8–13^. These observations suggest that the temporal fluctuation of *P*. *xylostella* populations is related to different climatic variables, such as temperature, rainfall and relative humidity^2,14,15^. Host-plant type does not seem to affect the population size of *P*. *xylostella*, but it affects its population dynamics^2,16^, resulting in a high variation of the abundance under limited plant availability^17^. Some studies used the CLIMEX model to predict the spatial and temporal distribution of *P*. *xylostella* and the frequency of its outbreaks^12,18^. However, there are few investigations that analyzed the spatio-temporal pattern of *P*. *xylostella* populations at the level of agricultural landscape, and little information is available about the moth’s distribution in habitats outside of cruciferous vegetable fields.

The dispersal capacity and survival of insects is related to landscape structure, such as landscape complexity, diversity, patch size, and fragmentation^19^. Landscape pattern in agroecosystems can greatly vary with both space and time. This variability has an important effect on the structure and dynamics of insect populations, and thus, leading to an impact on the distribution of insects^20–23^. *Plutella xylostella* populations are often unevenly distributed in different habitats and landscape, and exhibit a large variation^2,24^, but the underlying mechanisms are poorly understood. As an important statistical analysis tool in landscape ecology analysis, geostatistics which includes information from the geographical location of samples, is considered as a reliable technique to understand the spatial distribution of animals^25,26^.

Geographical information system (GIS) and geostatistics (GS) are both techniques particularly useful in investigations on the population distribution and dynamics of insects in agroecosystems that are stochastic and spatially structured^27,28^. Over the recent years, GIS and GS have been widely used to investigate the relationship between the dispersal patterns of pests and natural enemies in a fine scale of landscape^29,30^. The results can be used for improving strategies for better pest monitoring, prediction and management^31–33^.

Knowing about the temporal dynamics and spatial distribution of *P*. *xylostella* is fundamental for developing management programs to control this pest. It can provide important information to illustrate when and where *P*. *xylostella* should be controlled to avoid economic losses by formulating timely control measures. Therefore, in this study we used geostatistical methods to investigate the temporal dynamics and spatial distribution of the diamondback moth, *P*. *xylostella*, in a fine-scale agricultural landscape located in a coastal region near the Taiwan Strait, Southeast of China.

## Materials and methods

### Study area

**Figure 1:**
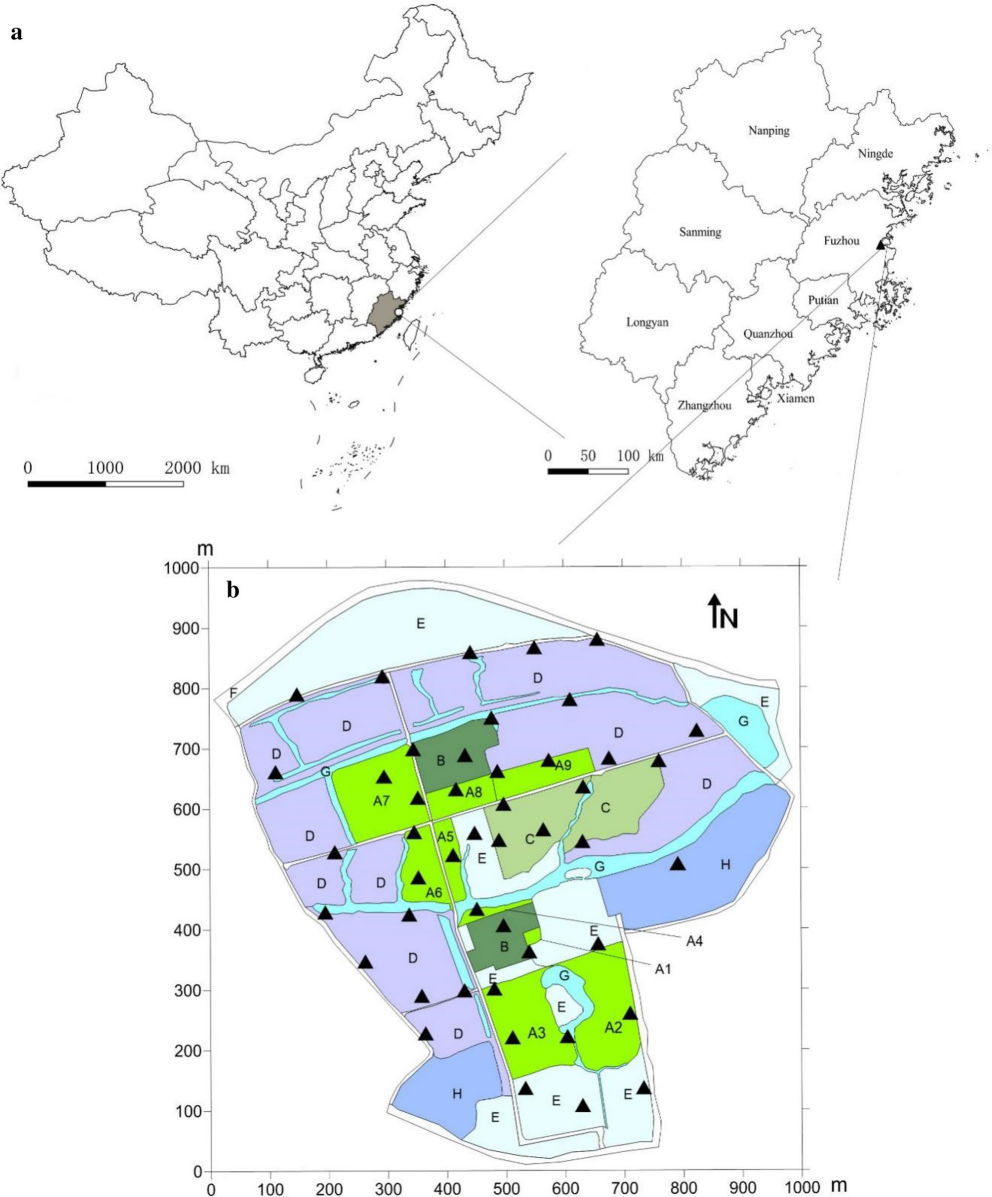
Geographical location and landscape composition of the study area. (**a**) The map was downloaded from the National Geomatics Center of China (http://www.ngcc.cn/ngcc/); (**b**) the base map was created using QGIS 3.0 based on the aerial photo of farm. A1–A9: Cruciferous vegetables grown in different patches (Table 1). B: Polytunnels, C: Rice fields, D: Pastures, E: Wastelands, F: Roads, G: Water bodies, H: Residential areas. Triangles (▲) represent traps placed in different patches within the study area.

The study was conducted on an organic vegetable farm located in the eastern coast of Fujian Province, China (Fig. 1a), with a total area of 84 ha. The farm is located in a sub-tropical region in the northern hemisphere (119° 31′ 40.14″ E, 26° 3′ 37.30″ N), characterized by mild winters (December to February), warm springs (March to June), hot and humid summers with irregular typhoons from July to September and warm autumns from October to November^34^. The farm is surrounded by a diverse landscape consisting of roads, water bodies, wastelands, polytunnels, pastures and both cruciferous and non-cruciferous crop fields (Fig. 1a, b). With the seasonally changing farming practices of intercrops cultivated in some of the patches, the landscape pattern varies over time (Fig. 1b; Table 1).

**Table 1:** Seasonal schedule for growing cruciferous vegetables in different patches of the landscape within the study area, from October 2015 to October 2017.

Patch	Growing season 1		Growing season 2		Growing season 3		Growing season 4	
	Cruciferous vegetables	Growing dates	Cruciferous vegetables	Growing dates	Cruciferous vegetables	Growing dates	Cruciferous vegetables	Growing dates
A1	*Brassica oleracea*	25 Dec. 2015–13 May 2016	*B. oleracea*	29 Aug. 2016–15 Nov. 2016	*B. napus*	15 Nov. 2016–17 Feb. 2017	*B. napus*	10 Mar. 2017–29 May 2017
A2	*Brassica pekinensis*	8 Jan. 2016–18 Feb. 2016	–		–		–	
A3	*Brassica napus*	25 Oct. 2015–28 Apr. 2016	–		*B. napus*	15 Nov. 2016–17 Feb. 2017	*B. napus*	10 Mar. 2017–29 May 2017
A4	*B. pekinensis*	8 Jan. 2016–18 Feb. 2016	*B. narinosa*	12 Apr. 2016–25 May 2016	*B. napus*	15 Nov. 2016–17 Feb. 2017	*B. napus*	10 Mar. 2017–29 May 2017
A5	*B. oleracea*	8 Jan. 2016–29 Apr. 2016	–		*B. pekinensis*	3 Nov. 2016–2 Dec. 2016	*B. oleracea*	10 Mar. 2017–9 Jun. 2017
A6	*B. pekinensis*	25 Dec. 2015–5 Mar. 2016	*B. pekinensis*	1 Apr. 2016–5 May 2016	*B. pekinensis*	3 Nov. 2016–2 Dec. 2016	*B. oleracea*	10 Mar. 2017–9 Jun. 2017
A7	*B. oleracea*	25 Oct. 2015–18 Feb. 2016	*Raphanus sativus*	21 May 2016–11 Jul. 2016	*B. pekinensis*	3 Nov. 2016–2 Dec. 2016	*B. napus*	10 Mar. 2017–29 May 2017
A8	–		–		*B. pekinensis*	22 Oct. 2016–25 Jan. 2017	*B. oleracea*	10 Mar. 2017–9 Jun. 2017
A9	*B. pekinensis*	25 Oct. 2015–25 Dec. 2015	*B. pekinensis*	22 Jan. 2016–1 Apr. 2016	*B. pekinensis*	22 Oct. 2016–2 Dec. 2016	*B. oleracea*	10 Mar. 2017–9 Jun. 2017

“–” represents non-cruciferous vegetables grown at the corresponding growing dates in the patch.

### Data collection

Forty-six pheromone-lure traps (Enjoy Wing trap, supplied by Zhangzhou Enjoy Agricultural Technology Co., Ltd.) were set up to catch *P. xylostella* in different patches within the study area, with 15 traps in the 9 patches of cruciferous vegetables, 4 in the vegetable fields inside polytunnels, 6 in the rice field patch, 12 in the patch of pastures, 4 in wastelands, 4 along roads, and 1 in a residential area (Fig. 1b). The chemical compound of the pheromone lure were cis-11-hexadecenyl acetate and trans-11-hexadecenyl acetate absorbed in natural rubber (red) core. The lures hung in the middle of the trap with green roof, 5 cm above a white sticky bottom of the trap. The traps were 30 cm above ground fixed at the top between two bamboo poles inserted into the ground. The distance between the traps ranged from 38 to 930 m. From 30 October 2015 to 17 September 2017, pheromone lures and white sticky plates were replaced fortnightly and the number of captured adult *P*. *xylostella* were recorded.

A handheld GPS (Garmin eTrex Legend, Taiwan) was used to set up a grid in the study area and to collate trap position data. The sampling area was divided into landscape elements and the trapping points were visualized using ArcGIS10.2 software (Environmental Systems Research Institute, ESRI 2013).

Meteorological data during the sampling period (from October 2015 to September 2017) were provided by the Fujian Meteorological Service Center^35^. The correlation between adult moth numbers and meteorological data (maximum temperature, minimum temperature, relative humidity and precipitation) was tested using the Pearson’s correlation coefficient (*P* = 0.05).

### Geostatistical analysis

Before spatial analysis, moth count data was log-transformed to approximate a normal distribution. The spatial dependence among *P. xylostella* samples was assessed based on these transformed data using semivariance analysis^28^. The semivariogram analysis was performed with the GS + software (Version 9, Gamma Design Software, Plainwell, MI, USA) for fortnightly counts of *P. xylostella*, provided that cumulated catches were greater than ten individuals (total 34 cases of 47 cases). Optimal models were fitted with the best fit being measured by the coefficient of determination (*R*^2^), residual sum of squares (RSS), *range* (*a*) and nugget (*C*_0_)^36^_._

At a certain distance, the semivariance stabilizes at a constant value. This constant semivariance is called the sill (C_0_ + *C*), this distance is the *range* (*a*), and the semivariance value at the intercept when the distance is equal to zero is called the nugget effect (*C*_0_)^37^. The C_0_/(C_0_ + *C*) ratio (level of spatial dependence, LSD) provides an estimation of the amount of randomness that exists in the data at spaces smaller than the sampling distance^30,38^. The spatial dependence of the semivariogram is considered strong when LSD ≤ 0.25, moderate when 0.25 < LSD ≤ 0.75, and weak when LSD > 0.75^30^.

Models obtained from the semivariogram analysis were used to interpolate *P. xylostella* catches by the means of the inverse distance weight method with the use of the squared values^33^. Spatial analyses were carried out using Surfer Version 14 (Golden software, Golden, CO, USA) with data columns *X*, *Y* representing latitude and longitude expressed as Universal Transversal Mercator coordinates, and *Z* representing the trap counts^28,37^. The obtained interpolation grid was graphically represented using a contour map layered on the base map of the experimental area^31^.

One-way ANOVA by SPSS statistics software was used to test the statistical differences between the yearly catches obtained from different landscape elements. Prior to the analysis, squareroot transformation ($\sqrt{x+1}$) was applied to normalize the distribution. The Tukey–Kramer test (*P* = 0.05) was used for multiple comparison, upon a significant difference obtained from the ANOVA.

## Results

### Temporal dynamics

During the sampling period 3543 *P*. *xylostella* males were collected in all traps set up in the study area. Based on the 2-year data of trap-captured specimens, we observed an early spring (March to April) peak of *P*. *xylostella* population, while summer and winter were less favorable, with a numerical decline of the population in these two seasons. *Plutella xylostella* population were significantly associated with the average daily minimum temperature (*P* = 0.002, *r* = − 0.450) and average daily maximum temperature (*P* = 0.011, *r* = − 0.370). There was no significant relationship between *P. xylostella* population size and relative humidity and average daily precipitation (*P* = 0.144, *r* = 0.216 and *P* = 0.781, *r* = − 0.042, respectively).

**Figure 2:**
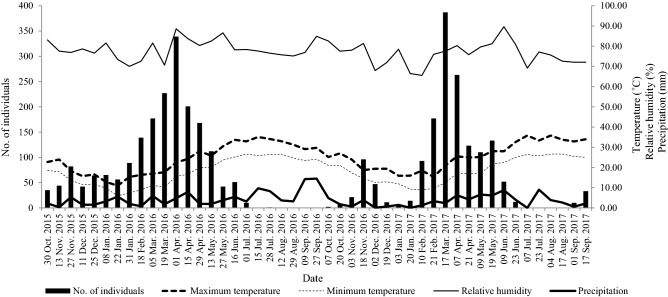
Trap catches of *P. xylostella* recorded every 2 weeks from October 2015 to September 2017. Average temperature minima, temperature maxima, mean relative humidity, and rainfall were measured every 2 weeks, with data being obtained from the daily recordings of a meteorological station located within the study area.

In the first year (from 30 October 2015 to 27 September 2016), 1944 males were trapped. The *P. xylostella* captures started in late October 2015, sharply increased in mid-February 2016, with the peak (339 males captured) on 10 April, and then declined until the middle of July, with no captures from 15 July to 27 September. In the second year (from 7 October 2016 to 17 September 2017), 1599 males were collected. Adult catches occurred at the end of October 2016, with a highest number of 387 males captured on 17 March 2017, and the catches gradually declined to no captures from 10 July to 17 August (Fig. 2).

### Spatial distribution

A total of the 47 semivariograms were calculated from the 2 years of sampling, and 34 mathematical models were successfully developed (Table 2). Of these, 19 cases were presented as Spherical models, and two cases (on 20 January 2016 and 10 February 2016) were shown as Gaussian models. These 21 population samples displayed strong spatial autocorrelation and aggregation. The remaining semivariograms did not result in an asymptotic model, indicating a random distribution.

**Table 2:** Spatial distribution patterns of *P. xylostella* populations in fine-scale agricultural landscape in different sampling time.

Sampling date	Number of individuals (n)	Model	Nugget *C*_0_	Stll (*C*_0_ + *C*)	Range *a* (m)	*R*^2^	Pattern
30 Oct. 2015	35	Nugget effect	0.343	0.343	–	–	Random
13 Nov. 2015	44	Spherical	0.070	0.540	84.85	0.48	Clumped
27 Nov. 2015	82	Spherical	0.053	0.770	90.30	0.08	Clumped
11 Dec. 2015	42	Nugget effect	0.338	0.338	-	-	Random
25 Dec. 2015	64	Spherical	0.018	0.514	102.10	0.09	Clumped
8 Jan. 2016	65	Spherical	0.008	0.541	157.20	0.72	Clumped
22 Jan. 2016	56	Spherical	0.025	0.453	113.20	0.21	Clumped
31 Jan. 2016	89	Spherical	0.073	0.701	82.66	0.48	Clumped
18 Feb. 2016	139	Nugget effect	0.819	0.819	-	-	Random
5 Mar. 2016	177	Spherical	0.147	1.216	93.33	0.80	Clumped
19 Mar. 2016	227	Spherical	0.105	1.244	82.00	0.38	Clumped
1 Apr. 2016	339	Spherical	0.168	1.301	96.42	0.97	Clumped
15 Apr. 2016	201	Spherical	0.135	1.461	81.21	0.47	Clumped
29 Apr. 2016	168	Spherical	0.094	1.186	99.22	0.12	Clumped
13 May 2016	112	Spherical	0.037	0.818	100.20	0.03	Clumped
27 May 2016	42	Nugget effect	0.479	0.479	–	–	Random
16 Jun. 2016	51	Nugget effect	0.594	0.594	–	–	Random
1 Jul. 2016	10	Nugget effect	0.175	0.175	–	–	Random
3 Nov. 2016	21	Spherical	0.088	0.341	90.20	0.89	Clumped
18 Nov. 2016	96	Spherical	0.131	1.019	92.66	0.77	Clumped
2 Dec. 2016	47	Nugget effect	0.552	0.552	–	–	Random
19 Dec. 2016	11	Nugget effect	0.133	0.133	–	–	Random
20 Jan. 2017	14	Gaussian	0.027	0.150	317.31	0.91	Clumped
10 Feb. 2017	93	Gaussian	0.068	0.675	130.94	0.59	Clumped
21 Feb. 2017	177	Spherical	0.098	1.313	111.19	0.57	Clumped
17 Mar. 2017	387	Spherical	0.114	1.647	84.70	0.08	Clumped
7 Apr. 2017	263	Spherical	0.151	1.766	118.30	0.62	Clumped
21 Apr. 2017	123	Spherical	0.146	1.113	85.89	0.61	Clumped
9 May 2017	110	Nugget effect	1.142	1.142	–	–	Random
19 May 2017	133	Nugget effect	1.199	1.199	–	–	Random
9 Jun. 2017	52	Nugget effect	0.625	0.625	–	–	Random
23 Jun. 2017	12	Nugget effect	0.182	0.182	–	–	Random
1 Sep. 2017	10	Nugget effect	0.156	0.156	–	–	Random
17 Sep. 2017	33	Spherical	0.084	0.458	98.62	0.70	Clumped

**Figure 3:**
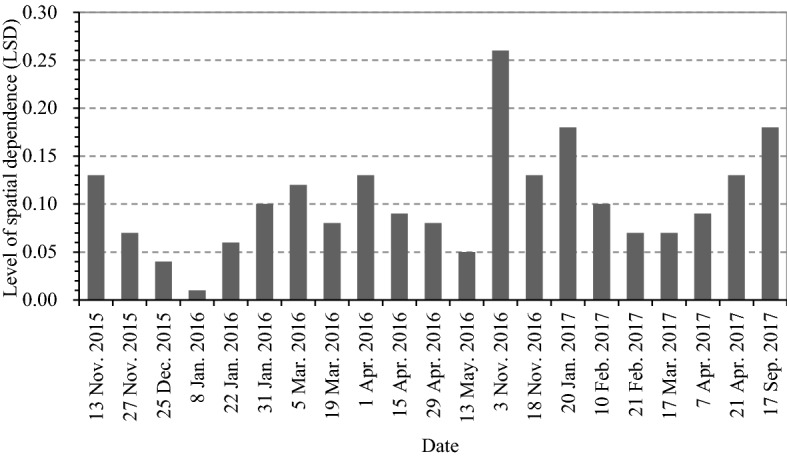
Spatial correlation data from *P. xylostella* populations produced by the semi-variance function model. We, Mo, and St represent weak, moderate, and strong spatial dependence, respectively.

The spherical and Gauss models showed small nugget values (0.008–0.168), large sill values (0.1498–1.766), strong spatial heterogeneity, and spatial variance ratios (*C*_0_/(*C*_0_ + *C*)) ranging from 0.01 to 0.26 (Fig. 3), which provided evidence for strong spatial autocorrelation. The spatial pattern of *P. xylostella* populations showed an aggregated distribution, with an estimated range from 82.00 to 317.31 m (Table 2). The 21 samples in the clumped pattern were collected during growth period (during which theleaf and harvestable vegetative plant parts develop in the cruciferous) vegetables, and comparatively, the samples collected during the crucifers’ maturity stage or when no crucifers were cultivated were randomly distributed.

### Contour maps of spatial distribution

**Figure 4:**
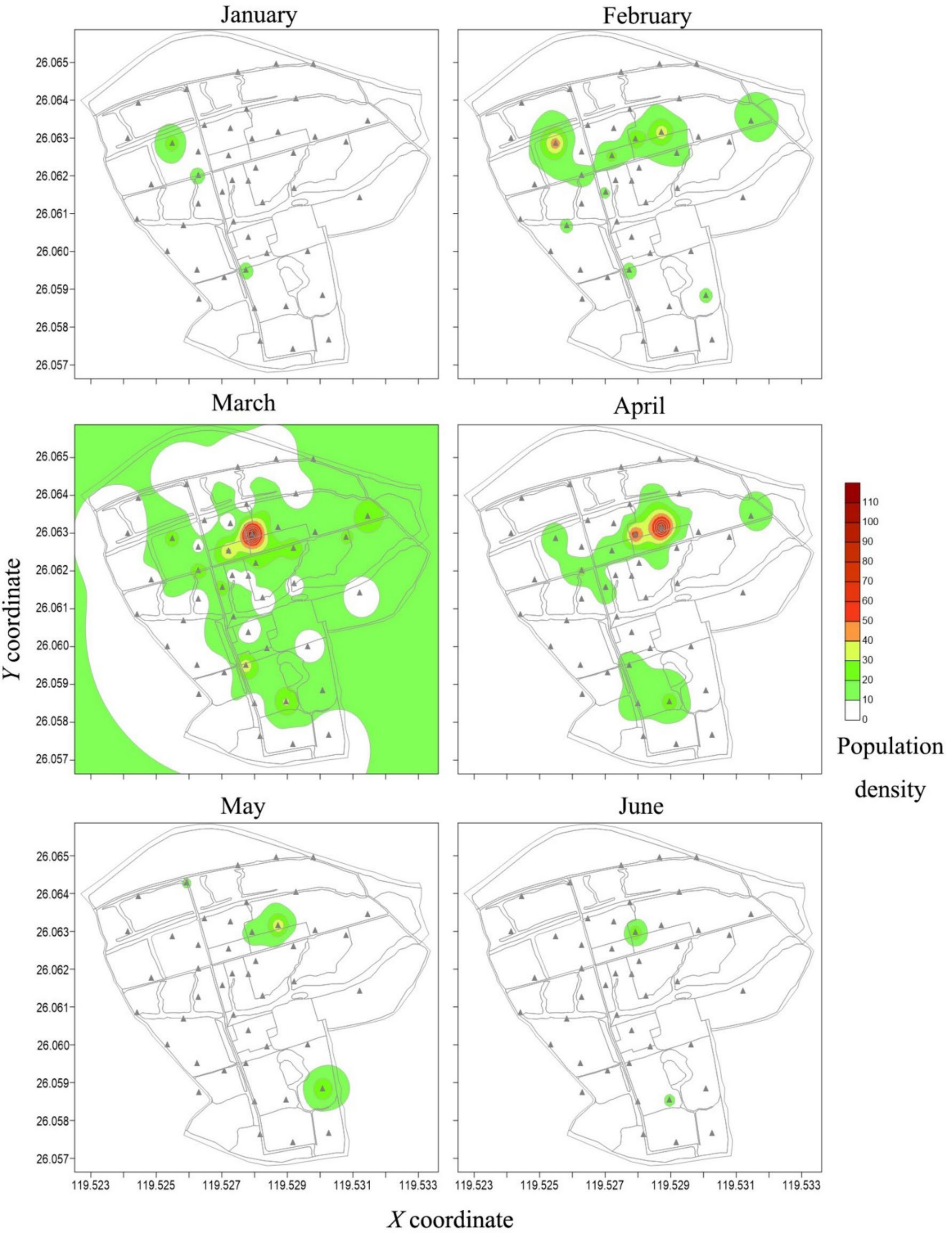
Contour maps of the *P. xylostella* distribution obtained by inverse distance squared weighted procedures applied to the monthly trap counts in 2016 using Surfer v14.0. Trap locations in the fields are shown with triangles (▲) the maps; *X* (longitude) and *Y* (latitude) axes are expressed in UTM coordinates.

**Figure 5:**
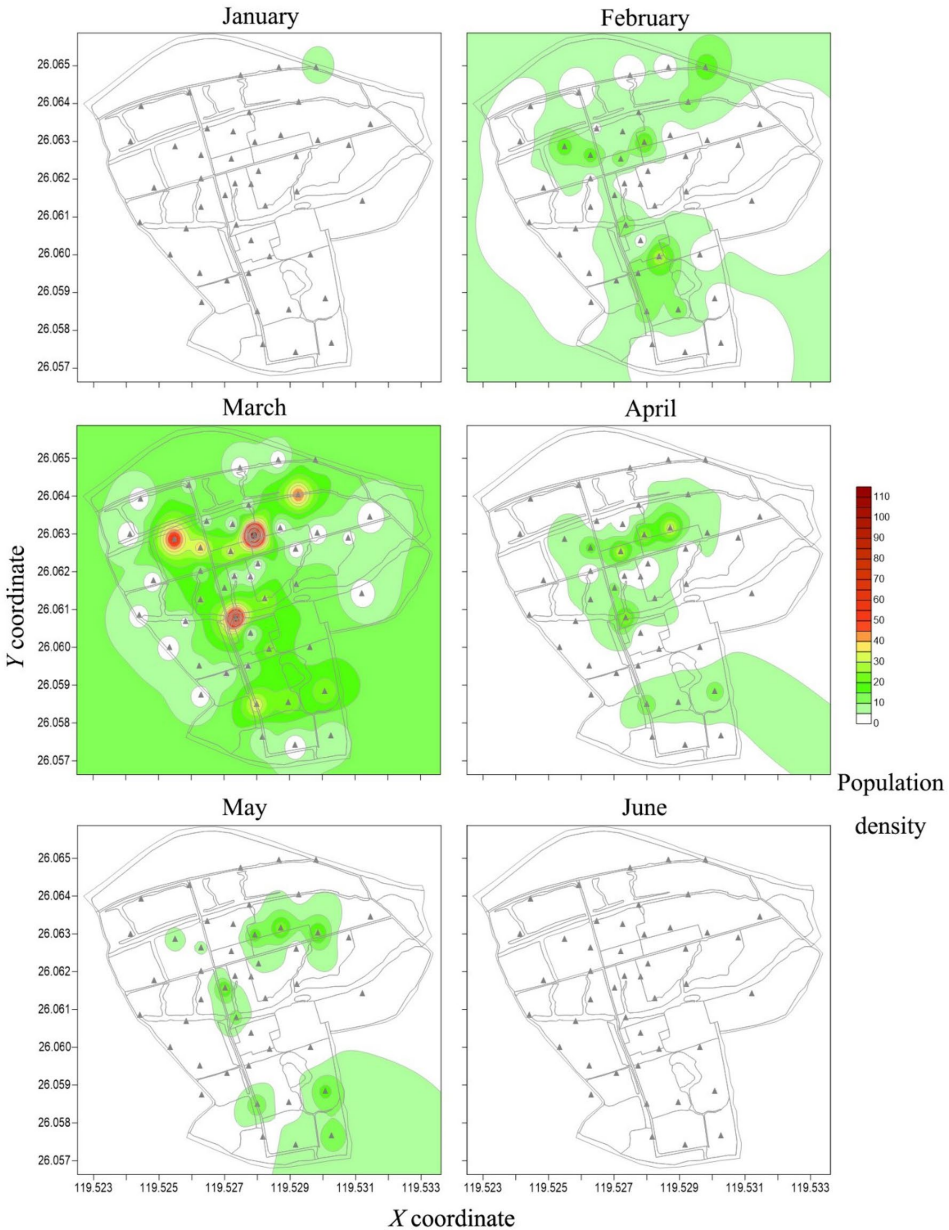
Contour maps of *P. xylostella* distribution obtained by inverse distance squared weighted procedures applied to the monthly trap counts in 2017 using Surfer v14.0. Trap location in the field is shown by triangles (▲); *X* (longitude) and *Y* (latitude) axes are expressed in UTM coordinates.

Two years contour maps created by the inverse distance squared weighted procedures exhibited a distribution pattern of *P. xylostella* in agricultural landscape (Figs. 4, 5). In January 2016, the *P. xylostella* population was relatively low, mostly inhabited in the patch of A7. Then *P. xylostella* population continued to increase from February to March, and spread widely on farms, with a marked peak in April and the main hotspot located in patch A9. After May, the population decreased, with a small hotspot being observed in patch A9, and then the population declined sharply with very low number of individuals trapped (Fig. 4).

In January 2017, *P. xylostella* adults were first found in the northeast of the experimental area, and then some small hotspots A1, A3, A4 and A7 were observed with the increase in the fields of cruciferous vegetables. In March, large hotspots began to appear in *B*. *oleracea* fields (A4, A5, A8 and A9), and this pattern of distribution continued to May. In late May, with the harvest of cruciferous vegetables, only a small number of *P. xylostella* could be attracted and no hotspots were observed in June (Fig. 5).

### Effect of landscape elements on the *P. xylostella* distribution

**Figure 6:**
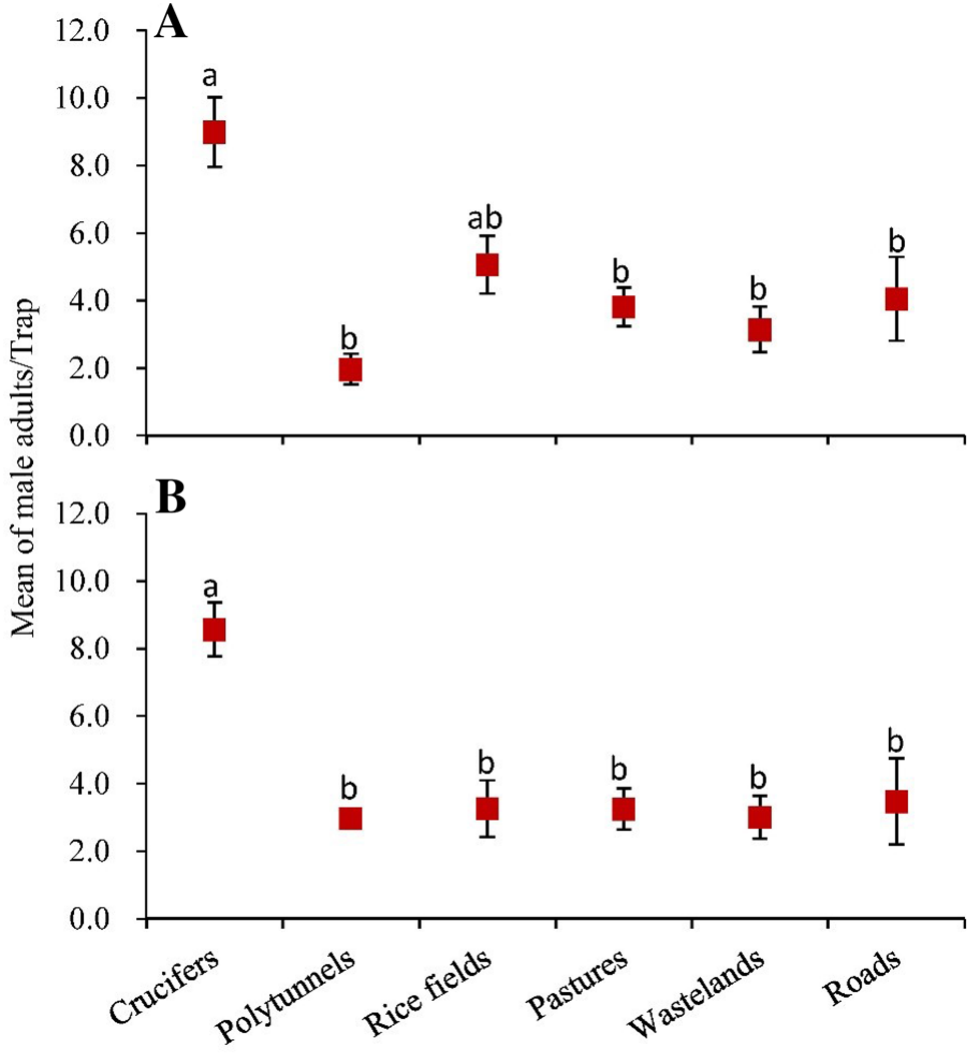
Mean number of *P*. *xylostella* adults (± SE) per trap during 2 years survey, collected on the main landscape elements where traps were positioned. Means with the same letter are not significantly different by Tukey–Kramer test (*P* = 0.05). (**A**) The first year (from 30 October 2015 to 27 September 2016), (**B**) The second year (from 7 October 2016 to 17 September 17 2017).

Significant differences were found in *P. xylostella* numbers between the landscape elements in both studied years (in the first year: *F*_5, 39_ = 7.402, *P* < 0.01; in the second year: *F*_5, 39_ = 9.776, *P* < 0.01). In the first year, traps positioned in cruciferous vegetable fields captured more *P. xylostella* adults, when compared with traps in polytunnels, pastures, wastelands or roads. However, the difference was not statistically significant compared to rice fields (Fig. 6A). In the second year, the number of *P. xylostella* adults caught in cruciferous vegetables was also significantly higher than those in other landscape elements (Fig. 6B). Traps in residential areas did not catch any adults of *P. xylostella*.

## Discussion

This study presents new information on the seasonal fluctuations of *P. xylostella* in a fine-scale agricultural landscape with different cropping and non-cropping areas, providing valuable contribution to the phenology of this destructive pest. More than 20 generations of *P. xylostella* can develop per year in south China, and chemical control is the main management strategy against them on cruciferous crops^38^. The characterization of the temporal dynamics and the spatial distribution of *P*. *xylostella* in the agricultural landscape provide important information for monitoring *P*. *xylostella* and assisting to develop effective pest management strategies targeting this pest. Although for management the presence of females is more important than that of males, and our baited traps mainly caught males, the general population patterns most likely can also be extrapolated to females.

The seasonal population dynamics and population peaks were apparent in the studied 2 years. The spring and autumn population peaks of *P. xylostella* in our study (Fig. 1) were consistent with previous reports^2,39,40^. In the southern regions of China (including Fuzhou), low temperatures in January and high temperatures in July and August are not favorable for *P*. *xylostella*^38,41,42^, thus number of captured individuals remained low in these months. In fact, the peaks of pheromone trap catches in November and March–April each year (Fig. 2) were well aligned with the largest presence of food crops (Table 1). Unlike other studies ^43–45^, we did not find significant relationships between *P*. *xylostella* population size and relative humidity or the average daily precipitation. This may be due to the long (fortnightly) sampling interval without heavy rain or long duration rainfall, resulting in no differences in population size. Another reason may be that the boat-type trap used in the experiment may have a rain-shielding effect.

Geostatistical analysis and semivariogram models exhibited spatial dependence in 21 of 47 samples in the agricultural landscape (i.e., spatial aggregation). Overall, the dispersion patterns of *P. xylostella* were aggregated during the growth periods of their hosts (from March to April 2016 and from January to April 2017) and random during the mature stage of the host plants (from May to July 2016 and from May to June 2017).

Contour maps indicated an aggregation of *P*. *xylostella* in the agricultural landscape, mainly synchronized with the availability of food plants in the area (patches of A7, A8 and A9; Figs. 4, 5), where cruciferous vegetables were grown. Individuals in some months were also located with low numbers, outside cabbage fields, most likely because moths were caught during their host searching flight. This varying response, reported also by other authors, was strongly related to the presence of cabbage^46,47^ and the dispersal pattern of *P*. *xylostella* population dynamics is associated with the shortage of favorable food^48^. When the crops were in their growth period (from March to April in 2016 and 2017), the ecological environment gradually stabilized and became more suitable for *P. xylostella*. The populations developed rapidly and stabilized in this period. While, at the mature stage of cruciferous vegetables (from May to July in 2016 and 2017), the deteriorated quality and the harvest of the crops is likely to be resulted in an unfavorable environment for the survival of *P. xylostella*^49^. Although the numbers of adult caught varied among patches, the trend of the population’s spatial distribution was highly similar in the two studied years, and the maps indicated that highest densities of *P*. *xylostella* were located in the areas of cruciferous vegetables. Therefore, the hot spots seemed to be linked not only to the species of host plants, but also to the growing stage of the plants influences the spatial distribution of *P*. *xylostella* population.

In recent years, significant attention has been paid to the impact of agricultural landscape on integrated pest management^50,51^. The observed pattern of *P*. *xylostella* distribution in our study increased towards the area of cruciferous vegetable growing, especially the cultivation of *Brassica* crops. *Plutella xylostella* captures were highly influenced by cropping systems at the regional level and the spatial trend of dispersion was consistent with the cabbage field^46^. In farmland ecosystems, *P*. *xylostella* showes a distinctive spatial distribution pattern among patches, and the layout of host plant patches is one of the drivers that affect this distribution pattern^5^.

Our results and similar studies of temporal dynamics and spatial patterns, as well as those use geostatistical analysis, can provide important information to develop a control measure in agricultural landscape. Cruciferous vegetables planting area is the main occurrence area of *P. xylostella*. Thus, one of the possible implications of this study for the management of *P*. *xylostella* is that a reasonable number of traps can be placed in and around cruciferous vegetable fields at the early growing stages of cruciferous vegetables to catch males to reduce mating and thus decrease population. Traps can be placed early in crucifers’ growth season to prevent further damage and this way, the use of pesticides can be minimized.

This study characterizes the temporal dynamics and the spatial distribution of *P. xylostella* in an agricultural landscape, and demonstrates that host distribution and growth stage may have a great impact on the spatial distribution of *P. xylostella* population. The results advance our understanding of temporal and spatial distribution of the *P. xylostella* population on a diversified farm in subtropical region, and provide knowledge of using pheromone baited traps and geostatistical analysis method as tools for monitoring and forecasting of the population dynamics and implementing the program of integrated pest management^52,53^.
